# Home-based Pilates for symptoms of anxiety, depression and fatigue among persons with multiple sclerosis: An 8-week randomized controlled trial

**DOI:** 10.1177/13524585211009216

**Published:** 2021-04-19

**Authors:** Karl M Fleming, Susan B Coote, Matthew P Herring

**Affiliations:** Physical Activity for Health Research Cluster, Health Research Institute, University of Limerick, Limerick, Ireland/Department of Physical Education and Sport Sciences, University of Limerick, Limerick, Ireland; Physical Activity for Health Research Cluster, Health Research Institute, University of Limerick, Limerick, Ireland/School of Allied Health, University of Limerick, and Multiple Sclerosis Society, Limerick, Ireland; Physical Activity for Health Research Cluster, Health Research Institute, University of Limerick, Limerick, Ireland/Department of Physical Education and Sport Sciences, University of Limerick, Limerick, Ireland

**Keywords:** Pilates, multiple sclerosis, anxiety, depression, fatigue

## Abstract

**Background:**

Symptoms of anxiety, depression and fatigue are common comorbidities among persons with multiple sclerosis (PwMS). A previous pilot study supported Pilates as a feasible exercise modality that may improve these outcomes among PwMS.

**Objective:**

To quantify the effects of 8 weeks of home-based Pilates on symptoms of anxiety, depression and fatigue among PwMS.

**Methods:**

A total of 80 PwMS (69 female) were randomized to twice-weekly home-based Pilates guided by a DVD) or wait-list control. Validated questionnaires assessed anxiety, depressive and fatigue symptoms at baseline, weeks 2, 4, 6 and 8. Using intention to treat, repeated measures analysis of covariance (RM-ANCOVA) adjusted for baseline physical activity examined between-group differences across time. Hedges’ *d* quantified the magnitude of differences in outcome change. Sensitivity analyses examined female-only samples.

**Results:**

Group × time interactions were statistically significant for all outcomes (all *p* ⩽ 0.005). Pilates significantly reduced (all *p* ⩽ 0.03) depressive symptoms (Quick Inventory of Depressive Symptomatology, *d* = 0.70; Hospital Anxiety and Depression Scale-Depression, *d* = 0.74), anxiety (State-Trait Anxiety Inventory, *d* = 0.30; Hospital Anxiety and Depression Scale-Anxiety, *d* = 0.49), cognitive (*d* = 0.44), physical (*d* = 0.78), psychosocial (*d* = 0.56) and total fatigue (*d* = 0.76). Female-only results were materially the same.

**Conclusion:**

Home-based Pilates significantly improved anxiety, depressive and fatigue symptoms among PwMS with minimal-to-mild mobility disability, including moderate-to-large, clinically meaningful improvements in depressive and fatigue symptoms.

Trial Registration: ClinicalTrials.gov (NCT04120207)

## Introduction

Multiple sclerosis (MS) is a chronic disease of the central nervous system characterized by inflammatory demyelination and axonal reduction.^1^ Despite growing evidence supporting benefits of exercise, people with MS (PwMS) are less active than healthy individuals.^1^ Among PwMS, symptoms of anxiety, depression and fatigue are prevalent,^2^ and traditional exercise modes improve these symptoms.^3,4^ However, the effects of non-traditional exercise, like Pilates, are understudied.

Pilates is a low-to-moderate intensity, mind–body exercise that improves core stability, muscular strength, flexibility, breathing and posture.^5^ It is easily learned and has elicited improved flexibility, dynamic balance and muscular endurance among healthy populations.^6^

Meta-analytic evidence supported moderate-to-large effects of supervised Pilates training on anxiety and depressive symptoms, energy and fatigue, and quality of life in healthy and chronically ill adults.^7^ Pilates controlled trials predominantly involve females, and evidence supports sex-related differences in mood responses to exercise.^8^ Conversely, limited evidence exists regarding effects of Pilates among PwMS, particularly home-based Pilates, which facilitates increased accessibility and engagement.^9^

Pilates’ floor-based approach addresses MS-specific considerations of overheating^10^ and fear of falling.^11^ Though positive effects on balance, muscular strength and functional mobility are established among PwMS,^12^ alternatives to enhance mental health among PwMS are needed. Pilates’ low-intensity stimulus offers an exercise option to address mental health comorbidities.

Process, resource, management and scientific feasibility metrics were supported in an 8-week randomized controlled trial (RCT) of supervised and home-based Pilates for symptoms of anxiety, depression and fatigue among women with MS with minimal-to-mild mobility disability^13^ that found full compliance, no attrition or reported adverse events for home-based Pilates. Acknowledging the small sample size, preliminary results supported moderate-to-large effects on symptoms of anxiety, depression and fatigue, particularly for home-based Pilates.^13^ These findings supported the feasibility of home-based Pilates to improve mental health outcomes among women with MS with minimal-to-mild mobility disability and warrant further investigation.

Thus, this RCT quantified the effects of 8-week home-based Pilates compared to wait-list control (WL) on anxiety, depressive and fatigue symptoms among PwMS. The authors hypothesized that Pilates would elicit significant moderate improvements in outcomes.

## Methods

### Trial design

This definitive, single-blind RCT compared home-based Pilates with a WL. The study protocol was approved by the University Research Ethics Committee and registered on ClinicalTrials.gov (NCT04120207). The content of the intervention is described in detail in the protocol paper.^14^ Participants provided written informed consent prior to participation. The Consolidated Standards of Reporting Trials checklist informed trial conduct and reporting.^15^

### Eligibility criteria

Inclusion criteria were: (1) adults (>18 years) with self-reported, physician-diagnosed MS; (2) patient-determined disease steps score < 3; (3) no conditions or medical contraindications that would preclude safely participating in a Pilates programme established with Physical Activity Readiness Questionnaire (PAR-Q); and (4) no previous Pilates experience. Exclusion criteria were (1) pregnancy, (2) MS relapse or (3) changes to MS medication or steroid treatment in prior 12 weeks.

### Interventions

The Pilates group performed twice weekly sessions, ~48 hours apart, for 8 weeks at home, supported by a DVD developed, implemented and evaluated in a feasibility trial among PwMS.^13^ Further intervention details can be found in the protocol paper.^14^ The DVD Pilates instructor is qualified with an experience of 10 years, does not have cognitive behavioral therapy (CBT), psychology or coaching training, but regularly teaches group classes to populations of various abilities. Compliance was monitored via exercise diaries containing session dates, completed exercise repetitions (four repetitions during first 2 weeks, increasing by two repetitions at biweekly intervals, resulting in 10 repetitions in the final 2 weeks) and session rating of perceived exertion (RPE), recorded by participants immediately following session completion. Participants were supported by a weekly telephone call entailing questions about frequency, intensity and duration of completed sessions, exercise completion difficulties, adverse events or relapses. WL maintained pre-intervention physical activity levels and were contacted by email or telephone to ensure completion of biweekly outcome assessments.

### Outcomes

Primary outcomes were anxiety, depressive and fatigue symptoms, for which validated measures were electronically administered (blind to the assessor) at baseline and every 2 weeks during the intervention, consistent with questionnaire recall timeframes. To improve comparability with previous investigations,^2–4,7,13^ multiple biweekly outcome assessments were completed. The 21-item Modified Fatigue Impact Scale (MFIS) assessed physical, cognitive, psychosocial, and total fatigue.^16^ Anxiety symptoms were measured with the 20-item trait subscale of the State-Trait Anxiety Inventory (STAI-Y2),^17^ and 7-item anxiety subscale of the Hospital Anxiety and Depression Scales (HADS-A).^18^ Depressive symptoms were assessed with the 16-item Quick Inventory of Depressive Symptomatology (QIDS)^19^ and 7-item depression subscale of the HADS (HADS-D).^18^ Physical activity was self-reported using a 7-day physical activity recall (7d-PAR),^20^ and Godin Leisure Time Exercise Questionnaire (GLTEQ).^21^ All measures were im-plemented and analysed in a feasibility study among PwMS with full compliance, confirming assessment procedures were appropriate/feasible.^13^

### Sample size

Based on effect sizes from our meta-analysis (anxiety (Δ = 1.29, 95% confidence interval (CI): 0.24, 2.33), depression (Δ = 1.27, 95% CI: 0.44, 2.09) and fatigue (Δ = 0.93, 95% CI: 0.21, 1.66)) and pilot study (*d* = 0.47–1.25; all *p* ⩽ 0.02),^7,13^ moderate-to-large magnitude differences in change in anxiety, depressive and fatigue symptoms following Pilates were hypothesized. Power analysis (G*Power) indicated a sample size of 56 would provide > 80% to detect a moderate-to-large effect (i.e. *f* = 0.325, *d* = 0.65), assuming a two-tail α = 0.05, four repeated measures and a conservative correlation between repeated measures. To account for potential 20% attrition among PwMS, a total of 68 were to be recruited to achieve the 28 participants per group.

### Recruitment

Recruitment began January 2018, and data collection ended August 2019. The home-based setting allowed national recruitment through MS Ireland via distribution of posters and participation information leaflets on social media and text alerts.^14^ Males and females were recruited to obtain a sample representative of the MS population in Ireland. The sample yielded an unequal gender distribution as expected given prevalence differences between males and females.^1^ Primary analysis used intention-to-treat in the full sample. Sensitivity analyses for the female-only sample are also included.

### Randomization

Following simple randomization procedures (computer-generated random numbers) using www.randomizer.org, eligible participants were randomized to Pilates or WL by an independent researcher not involved in outcome assessments. The lead author informed participants of allocation.

### Data monitoring

Study data, including recruitment numbers, eligibility, participant details and any participant issues (i.e. dropout, dropout reasons, relapses or adverse events), were monitored and recorded by the lead author in a password-protected central database. Outcome measures were completed electronically via www.surveymonkey.com and verified. The lead author contacted participants if measures were not completed.

### Statistical analyses

Analyses were conducted with IBM SPSS Statistics Version 25.0 (IBM Corp., Aramonk, NY). For intention-to-treat (ITT) analyses, conditional mean imputation was performed: age, gender, baseline physical activity and time-variant responses were entered into separate multiple linear regressions and predicted values were retained. Values for weeks 2–8, 4–8 and 6–8 were imputed for 10, 4 and 3 participants, respectively. Two group (Pilates, WL) by five time (baseline, weeks 2, 4, 6, 8) RM-ANCOVA adjusted for baseline physical activity examined between-group differences in outcomes across time. When sphericity was violated, the Huynh–Feldt adjustment was used. Significant interactions were decomposed with simple effects analysis, Bonferroni-corrected for multiple testing. Within-group magnitude of change and between-group magnitude of differences in change were quantified using standardized mean differences (*d*) and Hedges’ *d* (95% CIs), respectively.^22^ Improved outcomes and superiority of Pilates resulted in positive effect sizes. Consistent with Cohen’s suggestion, effect sizes of 0.2, 0.5 and 0.8 were judged as small, moderate and large, respectively.^23^ Using mean reduction in outcomes, number needed to treat (NNT) was calculated as a function of absolute risk reduction.^24^ Point-biserial correlations quantified associations between baseline severity and outcome change.

## Results

### Participant flow

**Figure 1.**
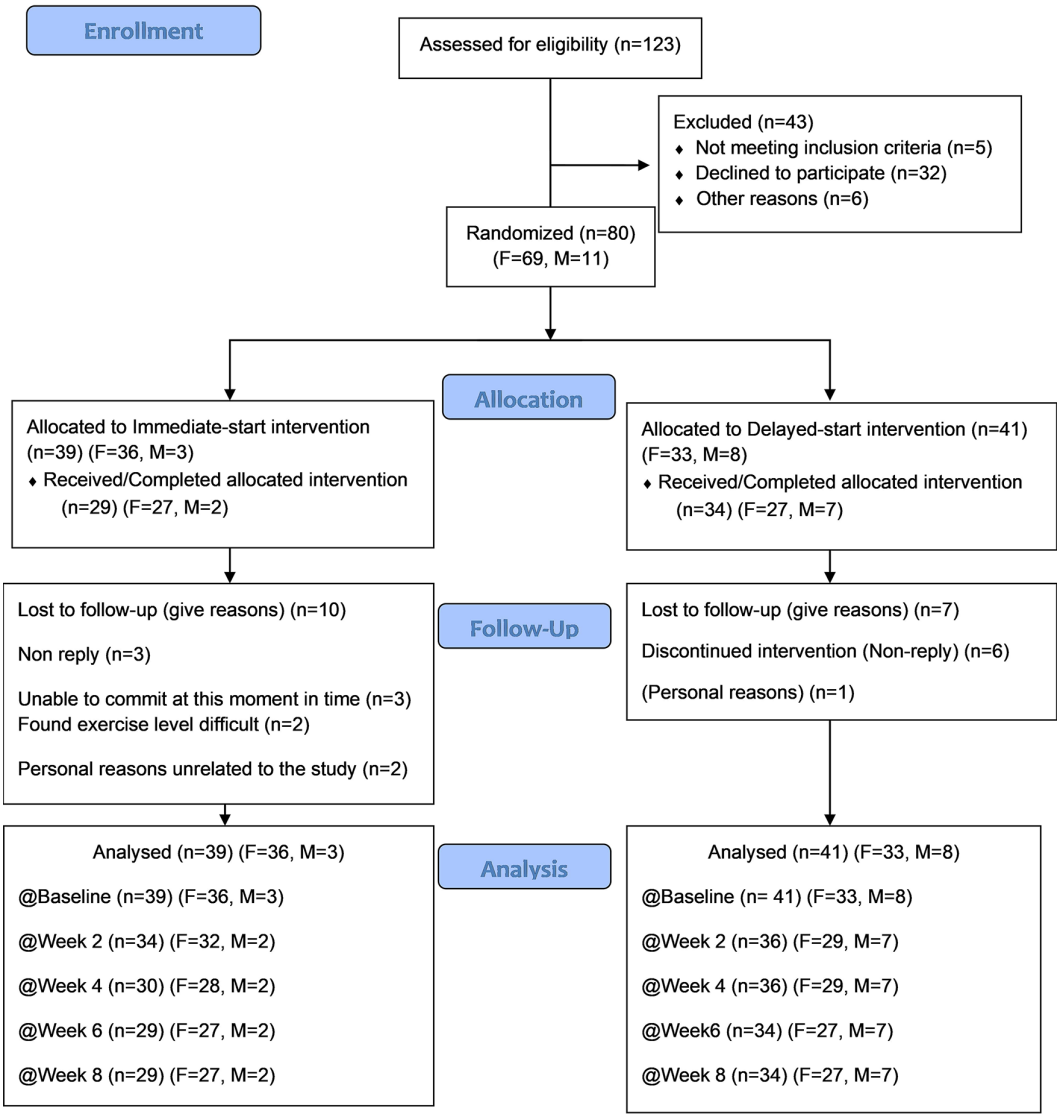
Participant flow through 8-week randomized controlled trial.

Figure 1 illustrates participant flow through the trial. Attrition was low and no adverse effects or relapses were reported.

### Baseline data

Table 1 presents baseline participant characteristics for the full ITT sample (*n* = 80). Successful randomization was supported by no baseline between-group differences in outcome variables. The 7d-PAR and weekly leisure activity (GLTEQ-WLA) were significantly higher in the WL group at baseline (both *p* < 0.04).

**Table 1.** Baseline participant characteristics.

Variable	ITT full sample			All completers			ITT female only			Female only completers		
	H/B (*n* = 39)	WL (*n* = 41)	Total (*n* = 80)	H/B (*n* = 29)	WL (*n* = 34)	Total (*n* = 63)	H/B F (*n* = 36)	WL F (*n* = 33)	Total (*n* = 69)	H/B (*n* = 27)	WL (*n* = 27)	Total (*n* = 54)
Age, years, mean (SD)	46.7 (10.0)	47.4 (10.2)	47.1 (10.0)	45.3 (8.6)	48.2 (9.76)	46.9 (9.3)	46.6 (9.6)	46.5 (11.0)	46.6 (10.2)	45.8 (8.6)	47.6 (10.7)	46.7 (9.6)
PDDS, *n* (%)												
Normal (0)	12 (30.8)	14 (34.1)	26 (32.5)	9 (31.0)	13 (38.2)	22 (34.9)	12 (33.3)	13 (39.4)	25 (36.2)	9 (33.3)	12 (44.4)	21 (38.9)
Mild disability (1)	9 (23.1)	6 (14.6)	15 (18.8)	8 (27.6)	5 (14.7)	13 (20.6)	8 (22.2)	5 (15.2)	13 (18.8)	7 (25.9)	4 (14.8)	11 (20.4)
Moderate disability (2)	3 (7.7)	2 (4.9)	5 (6.3)	3 (10.3)	2 (5.9)	5 (7.9)	3 (8.3)	1 (3.0)	4 (5.8)	3 (11.1)	1 (3.7)	4 (7.4)
Gait disability (3)	15 (38.5)	19 (46.3)	34 (42.5)	9 (31.0)	14 (41.2)	23 (36.5)	13 (36.1)	14 (42.4)	27 (39.1)	8 (29.6)	10 (37.0)	18 (33.3)
Depression												
QIDS	8.7 ± 4.1	7.8 ± 4.9	8.3 ± 4.5	8.7 ± 4.2	7.5 ± 4.7	8.1 ± 4.5	9.0 ± 4.1	8.5 ± 5.1	8.8 ± 4.6	8.8 ± 4.3	8.2 ± 4.9	8.5 ± 4.6
Severity, *n* (%)												
None (0–5)	7 (17.9%)	17 (41.5%)	24 (30.0%)	6 (20.7%)	14 (41.2%)	20 (31.7%)	6 (16.7%)	11 (33.3%)	17 (24.6%)	6 (22.2%)	9 (33.3%)	15 (27.8%)
Mild (6–10)	20 (51.3%)	12 (29.3%)	32 (40.0%)	15 (51.7%)	11 (32.4%)	26 (41.3%)	18 (50.0%)	11 (33.3%)	29 (42.0%)	13 (48.1%)	10 (37.0%)	23 (42.6%)
Moderate (11–15)	8 (20.5%)	8 (19.5%)	16 (20.0%)	5 (17.2%)	6 (17.6%)	11 (17.5%)	8 (22.2%)	7 (21.2%)	15(21.7%)	5 (18.5%)	5 (18.5%)	10 (18.5%)
Severe (16–20)	4 (10.3%)	4 (9.8%)	8 (10.0%)	3 (10.3%)	3 (8.8%)	6 (9.5%)	4 (11.1%)	4 (12.1%)	8 (11.6%)	3 (11.1%)	3 (11.1%)	6 (11.1%)
HADS-D	6.8 ± 3.3	5.7 ± 3.1	6.2 ± 3.2	7.1 ± 3.5	5.3 ± 3.2	6.1 ± 3.4	6.9 ± 3.2	5.8 ± .3.3	6.4 ± 3.3	7.0 ± 3.6	5.4 ± 3.3	6.2 ± 3.5
Severity, *n* (%)												
Normal (0–7)	25 (64.1%)	30 (73.2%)	55 (68.8%)	18 (62.1%)	27 (79.4%)	45 (71.4%)	23 (63.9%)	24 (72.7%)	47 (68.1%)	17 (63.0%)	22 (81.5%)	39 (72.2%)
Borderline (8–10)	11 (28.2%)	6 (14.6%)	17 (21.3%)	8 (27.6%)	3 (8.8%)	11 (17.5%)	11 (30.6%)	6 (18.2%)	17 (24.6%)	8 (29.6%)	3 (11.1%)	11 (20.4%)
Abnormal case (11–21)	3 (7.7%)	5 (12.2%)	8 (10.0%)	3 (10.3%)	4 (11.8%)	7 (11.1%)	2 (5.6%)	3 (9.1%)	5 (7.2%)	2 (7.4%)	2 (7.4%)	4 (7.4%)
Anxiety												
STAI-Y2	43.0 ± 9.8	41.3 ± 11.8	42.1 ± 10.8	43.8 ± 10.5	40.8 ± 11.9	42.2 ± 11.3	43.1 ± 9.8	43.4 ± 11.3	43.3 ± 10.5	43.4 ± 10.8	42.9 ± 11.4	43.2 ± 11.0
High trait anxious (> 50), *n* (%)	5 (12.8%)	8 (19.5%)	13 (16.3%)	4 (13.8%)	7 (20.6%)	11 (17.5%)	5 (13.9%)	8 (24.2%)	13 (18.8%)	4 (14.8%)	7 (25.9%)	11 (20.4%)
Non-high trait anxious (< 50), *n* (%)	34 (87.2%)	33 (80.5%)	67 (83.8%)	25 (86.2%)	27 (79.4%)	52 (82.5%)	31 (86.1%)	25 (75.8%)	56 (81.2%)	23 (85.2%)	20 (74.1%)	43 (79.6%)
HADS-A	8.4 ± 4.1	7.0 ± 4.3	7.7 ± 4.2	8.7 ± 4.0	6.9 ± 4.0	7.7 ± 4.0	8.5 ± 3.8	7.6 ± 4.5	8.1 ± 4.1	8.6 ± 3.8	7.3 ± 4.1	7.9 ± 4.0
Severity, *n* (%)												
Normal (0–7)	17 (43.6%)	24 (58.5%)	41 (51.2%)	12 (41.4%)	20 (58.8%)	32 (50.8%)	15 (41.7%)	17 (51.5%)	32 (46.4%)	11 (40.7%)	14 (51.9%)	25 (46.3%)
Borderline (8–10)	8 (20.5%)	10 (24.4%)	18 (22.5%)	6 (20.7%)	9 (26.5%)	15 (23.8%)	8 (22.2%)	9 (27.3%)	17 (24.6%)	7 (25.9%)	8 (29.6%)	15 (27.8%)
Abnormal case (11–21)	14 (35.9%)	7 (17.1%)	21 (26.3%)	11 (37.9%)	5 (14.7%)	16 (25.4%)	13 (36.1%)	7 (21.2%)	20 (29.0%)	9 (33.3%)	5 (18.5%)	14 (25.9%)
Fatigue												
MFIS total	43.6 ± 9.8	43.6 ± 14.3	43.6 ± 12.2	43.9 ± 9.5	42.0 ± 15.1	42.9 ± 12.8	44.0 ± 9.9	44.2 ± 14.7	44.1 ± 12.3	43.8 ± 9.9	42.5 ± 15.7	43.2 ± 13.0
Fatigued (> 38), *n* (%)	27 (69.2%)	28 (68.3%)	55 (68.8%)	21 (72.4%)	21 (61.8%)	42 (66.7%)	25 (69.4%)	23 (69.7%)	48 (69.6%)	8 (29.6%)	10 (37%)	18 (33.3%)
Non-fatigued (< 38), *n* (%)	12 (30.8%)	13 (31.7)	25 (31.3%)	8 (27.6%)	13 (38.2%)	21 (33.3%)	11 (30.6%)	10 (30.3%)	21 (30.4%)	19 (70.4%)	17 (63%)	36 (66.7%)
MFIS PHYS	22.1 ± 5.5	22.3 ± 7.1	22.2 ± 6.3	21.9 ± 5.6	21.9 ± 7.4	21.9 ± 6.6	22.1 ± 5.4	21.8 ± 7.0	22.0 ± 6.2	21.7 ± 5.5	21.6 ± 7.3	21.7 ± 6.4
MFIS COGN	16.8 ± 7.1	17.1 ± 7.8	17.0 ± 7.4	17.5 ± 5.6	16.0 ± 7.9	16.7 ± 6.9	17.3 ± 6.8	18.1 ± 7.9	17.7 ± 7.3	17.7 ± 5.3	16.9 ± 8.0	17.3 ± 6.7
MFIS PSYCH	4.6 ± 1.5	4.3 ± 2.3	4.5 ± 1.9	4.5 ± 1.5	4.1 ± 2.4	4.3 ± 2.0	4.6 ± 1.4	4.3 ± 2.3	4.4 ± 1.9	4.4 ± 1.4	4.0 ± 2.3	4.2 ± 1.9
Physical activity												
GLTEQ–total	20.5 ± 17.2	30.8 ± 25.2	25.8 ± 22.1	24.5 ± 17.6	32.4 ± 25.4	28.8 ± 22.4	21.6 ± 17.2	29.6 ± 21.3	25.4 ± 19.6	25.5 ± 17.6	30.2 ± 21.1	27.9 ± 19.4
PA-MOD	4.2 ± 3.0	7.1 ± 5.7	5.6 ± 4.8	4.4 ± 3.1	6.4 ± 4.9	5.5 ± 4.2	4.1 ± 3.0	7.8 ± 5.7	5.8 ± 4.9	4.5 ± 3.2	6.9 ± 4.9	5.7 ± 4.3
PA-HARD	1.1 ± 2.1	1.6 ± 3.2	1.4 ± 2.7	1.1 ± 2.3	1.7 ± 3.4	1.5 ± 3.0	1.1 ± 2.2	1.3 ± 3.0	1.2 ± 2.6	1.2 ± 2.4	1.4 ± 3.2	1.3 ± 2.8
PA-VHARD	0.2 ± 0.5	0.6 ± 1.4	0.4 ± 1.1	0.2 ± 0.4	0.6 ± 1.5	0.5 ± 1.2	0.3 ± 0.5	0.7 ± 1.6	0.5 ± 1.1	0.2 ± 0.4	0.8 ± 1.7	0.5 ± 1.3

ITT: intention to treat; H/B: home-based intervention group; WL: wait-list control; PDDS: patient-determined disease steps score; QIDS: Quick Inventory of Depressive Symptomatology; HADS-D: Depression Subscale of the Hospital Anxiety and Depression Scale; STAI-Y2: Trait Subscale of the State-Trait Anxiety Inventory; HADS-A: Anxiety Subscale of the Hospital Anxiety and Depression Scale; MFIS total: Modified Fatigue Impact Scale total score; MFIS PHYS: Physical Subscale of the Modified Fatigue Impact Scale; MFIS COGN: Cognitive Subscale of the Modified Fatigue Impact Scale; MFIS PSYCH: Psychosocial Subscale of the Modified Fatigue Impact Scale; GLTEQ–Total: Godin Leisure Time Exercise Questionnaire total leisure activity score; PA-MOD: moderate physical activity category of the seven-day physical activity recall (7d-PAR); PA-HARD: hard physical activity category of the 7d-PAR; PA-VHARD: very hard physical activity category of the 7d-PAR.

There were no statistically significant differences between ITT and completers, and between ITT female only and female only completers at baseline (All *p* > 0.48). The 7d-PAR and weekly leisure activity (GLTEQ-WLA) were significantly higher in the WL group (All completers) at baseline (both *p* < 0.04), but there were no significant differences between groups across time.

### Outcomes and estimations

**Figure 2.**
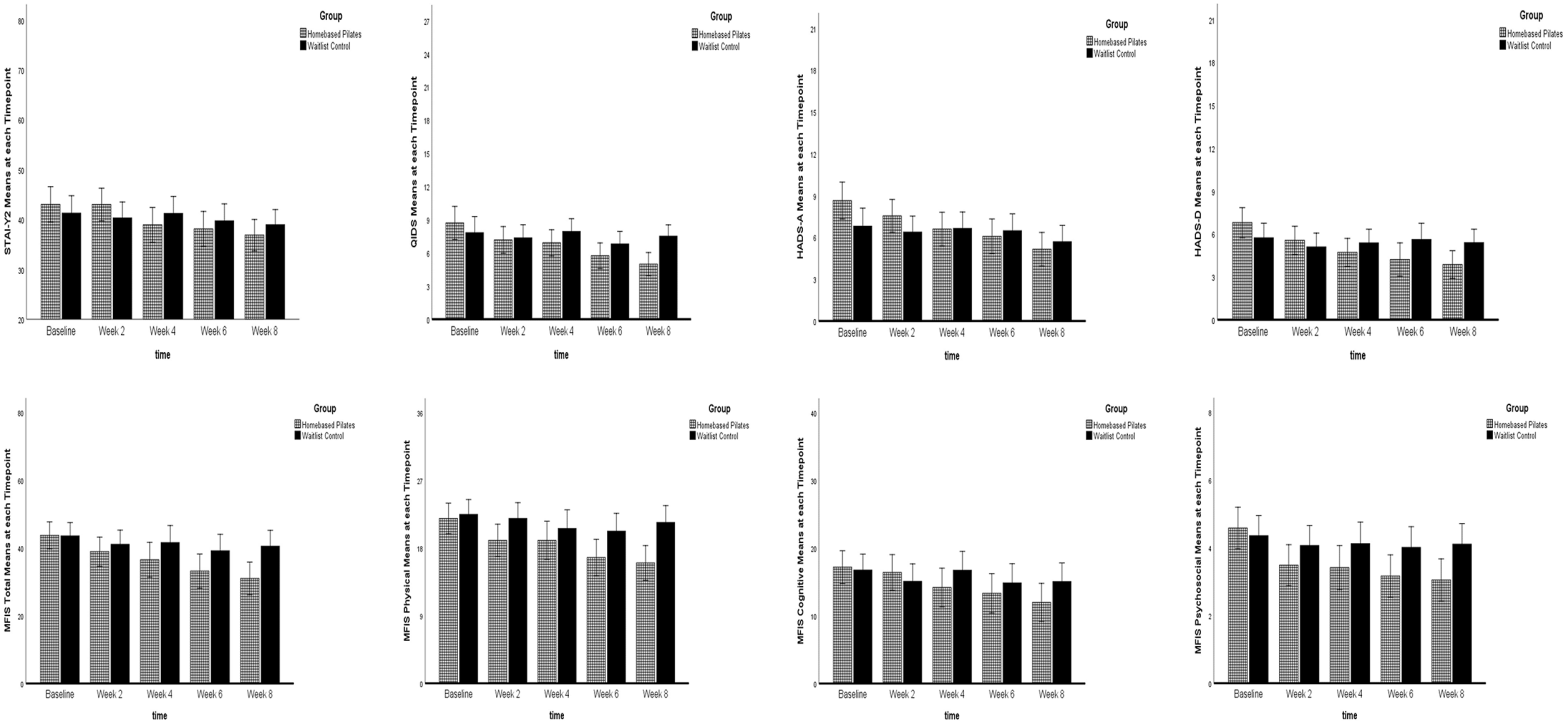
STAI-Y2 (0–80), QIDS (0–27), HADS-A (0–21), HADS-D (0–21), MFIS total (0–84), MFIS physical (0–36), MFIS cognitive (0–40), MFIS psychosocial (0–8) means at each timepoint.

Table 2 presents descriptives and effect sizes for the full ITT sample (*n* = 80). Table 3 presents descriptives and effect sizes for the completer sample (*n* = 63). Figure 2 illustrates mean changes for outcomes across time. Supplementary Tables 1 and 2 present descriptives and effect sizes for female-only ITT (*n* = 69) and completer (*n* = 54) samples. Supplementary Tables 3 and 4 present baseline symptom severity classifications and associations between baseline symptom severity and outcome change, respectively. Higher baseline severity was associated with greater outcome change (*r*_pb_ = 0.13–0.76). Mean session RPE across the 16 sessions for the home-based completers was 12 ± 1.

**Table 2.** ITT primary outcome changes at each measurement for overall sample – means (SD), within-group magnitude of change and between-group magnitude of differences in change were quantified using standardized mean differences (*d*) and Hedges’ *d* (95% CIs), respectively).

Outcome	Baseline	WK 2			WK 4			WK 6			WK 8		
	Mean ± SD	Mean ± SD	*d*	Hedges’ *d* from baseline	Mean ± SD	*d*	Hedges’ *d* from baseline	Mean ± SD	*d*	Hedges’ *d* from baseline	Mean ± SD	*d*	Hedges’ *d* from baseline
STAI–Y2													
Intervention	43.0 ± 9.8	43.1 ± 9.2	−0.01	−0.10 (−0.54, 0.34)	39.2 ± 9.6^a^	0.39	0.32 (−0.12, 0.76)	38.1 ± 10.1^a^	0.50	0.30 (−0.14, 0.74)	37.1 ± 9.1^a^	0.60	0.30 (−0.14, 0.74)
Control	41.3 ± 11.8	40.3 ± 10.7	0.08		41.0 ± 11.6	0.03		39.7 ± 11.2	0.14		38.7 ± 10.2	0.22	
HADS–A													
Intervention	8.4 ± 4.1	7.3 ± 3.6	0.27	0.16 (−0.27, 0.60)	6.6 ± 3.4^a^	0.44	0.35 (−0.09, 0.80)	6.0 ± 3.1^a^	0.59	0.45 (0.00, 0.89)	5.1 ± 3.0^a^	0.80	**0.49 (0.05**, **0.94)**
Control	7.0 ± 4.3	6.6 ± 3.9	0.09		6.7 ± 4.0	0.07		6.5 ± 4.4	0.12		5.8 ± 4.3	0.28	
QIDS													
Intervention	8.7 ± 4.1	7.1 ± 3.5	0.39	0.28 (−0.16, 0.73)	6.9 ± 3.2	0.44	0.42 (−0.03, 0.86)	5.8 ± 3.6^a^	0.71	0.42 (−0.03, 0.86)	5.1 ± 2.7^a,b^	0.88	**0.70 (0.25**, **1.15)**
Control	7.8 ± 4.9	7.5 ± 3.9	0.06		7.9 ± 4.0	−0.02		6.8 ± 3.5	0.20		7.4 ± 3.7	0.08	
HADS–D													
Intervention	6.8 ± 3.3	5.6 ± 3.0^a^	0.36	0.19 (−0.25, 0.63)	4.8 ± 2.7^a^	0.61	**0.50 (0.05**, **0.94)**	4.2 ± 3.1^a^	0.79	**0.77 (0.32, 1.23)**	4.0 ± 3.1^a,b^	0.85	**0.74 (0.29**, **1.20)**
Control	5.7 ± 3.1	5.1 ± 3.0	0.19		5.3 ± 3.3	0.13		5.6 ± 3.9	0.03		5.3 ± 3.0	0.13	
MFIS total													
Intervention	43.6 ± 9.8	38.6 ± 11.9^a^	0.51	0.23 (−0.21, 0.66)	36.5 ± 14.5^a^	0.72	0.41 (−0.03, 0.85)	33.3 ± 13.6^a^	1.05	**0.46 (0.01**, **0.90)**	31.0 ± 13.5^a,b^	1.29	**0.76 (0.31**, **1.22)**
Control	43.6 ± 14.3	41.4 ± 14.3	0.15		41.6 ± 16.7	0.14		39.0 ± 16.8	0.32		40.5 ± 15.8	0.22	
MFIS PHYS													
Intervention	22.1 ± 5.5	19.1 ± 5.1^a^	0.55	0.39 (−0.05, 0.83)	19.0 ± 6.4^a^	0.56	0.22 (−0.22, 0.66)	16.9 ± 6.6^a,b^	0.95	**0.45 (0.01, 0.89)**	16.1 ± 6.2^a,b^	1.09	**0.78 (0.32**, **1.23)**
Control	22.3 ± 7.1	21.8 ± 7.7	0.07		20.6 ± 8.9	0.24		20.0 ± 8.3	0.32		21.3 ± 7.9	0.14	
MFIS COGN													
Intervention	16.8 ± 7.1	16.0 ± 8.4	0.11	−0.11 (−0.54, 0.33)	14.1 ± 8.9	0.38	0.33 (−0.11, 0.77)	13.1 ± 8.6^a^	0.52	0.23 (−0.21, 0.67)	11.7 ± 8.3^a^	0.72	0.44 (−0.01, 0.88)
Control	17.1 ± 7.8	15.5 ± 8.1	0.21		16.9 ± 8.5	0.03		15.1 ± 9.1	0.26		15.3 ± 9.0	0.23	
MFIS PSYCH													
Intervention	4.6 ± 1.5	3.5 ± 1.6^a^	0.73	0.41 (−0.04, 0.85)	3.5 ± 1.7^a^	0.73	**0.46 (0.01**, **0.90)**	3.3 ± 1.8^a^	0.87	**0.46 (0.01**, **0.90)**	3.2 ± 1.8^a,b^	0.93	**0.56 (0.11**, **1.01)**
Control	4.3 ± 2.3	4.0 ± 2.1	0.13		4.1 ± 2.3	0.09		3.9 ± 2.2	0.17		4.0 ± 2.2	0.13	

ITT: intention to treat; CI: confidence interval; WK: week; STAI-Y2: Trait Subscale of the State-Trait Anxiety Inventory; HADS-A: Anxiety Subscale of the Hospital Anxiety and Depression Scale; QIDS: Quick Inventory of Depressive Symptomatology; HADS-D: Depression Subscale of the Hospital Anxiety and Depression Scale; MFIS total: Modified Fatigue Impact Scale total score; MFIS PHYS: Physical Subscale of the Modified Fatigue Impact Scale; MFIS COGN: Cognitive Subscale of the Modified Fatigue Impact Scale; MFIS PSYCH: Psychosocial Subscale of the Modified Fatigue Impact Scale.

Bold Hedges’ *d* effect sizes are statistically significant based on 95% CI not encompassing 0.

a A statistically significant difference from baseline (*p* < 0.05).

b A statistically significant difference from Control (p < 0.05).

**Table 3.** Primary outcome changes at each measurement – means (SD), within-group magnitude of change and between-group magnitude of differences in change were quantified using standardized mean differences (*d*) and Hedges’ *d* (95% CIs), respectively) (completer-only).

Outcome	Baseline	WK 2			WK 4			WK 6			WK 8		
	Mean ± SD	Mean ± SD	*d*	Hedges’ *d* from baseline	Mean ± SD	*d*	Hedges’ *d* from baseline	Mean ± SD	*d*	Hedges’ *d* from baseline	Mean ± SD	*d*	Hedges’ *d* from baseline
STAI-Y2													
Intervention	43.8 ± 10.5	43.3 ± 9.4	0.05	0.02 (−0.51, 0.48)	39.9 ± 10.5	0.37	0.34 (−0.16, 0.84)	38.6 ± 11.1^a^	0.50	0.35 (−0.15, 0.85)	37.2 ± 10.0^a^	0.63	0.38 (–0.12, 0.88)
Control	40.8 ± 11.9	40.1 ± 11.4	0.06		40.8 ± 12.5	0.00		39.6 ± 11.9	0.10		38.5 ± 11.0	0.19	
HADS–A													
Intervention	8.7 ± 4.0	7.3 ± 3.3	0.35	0.25 (−0.25, 0.74)	6.7 ± 3.6^a^	0.50	0.42 (−0.08, 0.92)	6.1 ± 3.0^a^	0.65	**0.52 (0.01**, **1.02)**	5.1 ± 2.8^a^	0.90	**0.59 (0.09**, **1.09)**
Control	6.9 ± 4.0	6.5 ± 4.0	0.10		6.6 ± 4.1	0.08		6.4 ± 4.6	0.12		5.7 ± 4.5	0.30	
QIDS													
Intervention	8.7 ± 4.2	7.2 ± 3.3	0.36	0.26 (−0.23, 0.76)	7.0 ± 3.7	0.40	0.44 (−0.06, 0.94)	5.7 ± 4.1^a^	0.71	0.49 (−0.02, 0.99)	5.0 ± 3.1^a,b^	0.88	**0.75 (0.24**, **1.26)**
Control	7.5 ± 4.7	7.2 ± 4.0	0.06		7.8 ± 4.3	−0.06		6.7 ± 3.8	0.17		7.2 ± 4.0	0.06	
HADS–D													
Intervention	7.1 ± 3.5	5.8 ± 3.2^a^	0.37	0.27 (−0.23, 0.76)	5.0 ± 3.0^a^	0.60	**0.53 (0.03**, **1.04)**	4.5 ± 3.4^a^	0.74	**0.77 (0.26**, **1.28)**	4.0 ± 3.5^a^	0.89	**0.86 (0.34**, **1.37)**
Control	5.3 ± 3.2	4.9 ± 3.2	0.12		5.0 ± 3.5	0.09		5.3 ± 4.2	0.00		5.1 ± 3.2	0.06	
MFIS total													
Intervention	43.9 ± 9.5	39.3 ± 12.3	0.48	0.22 (−0.27, 0.72)	36.3 ± 15.9^a^	0.80	0.48 (−0.03, 0.98)	33.3 ± 14.7^a^	1.12	0.49 (−0.01, 1.00)	31.1 ± 14.9^a,b^	1.35	**0.80 (0.29**, **1.31)**
Control	42.0 ± 15.1	40.3 ± 15.3	0.11		40.6 ± 18.1	0.09		37.8 ± 18.0	0.28		39.6 ± 17.1	0.16	
MFIS PHYS													
Intervention	21.9 ± 5.6	19.2 ± 5.6^a^	0.48	0.39 (−0.11, 0.89)	18.7 ± 7.1	0.57	0.21 (−0.29, 0.71)	16.7 ± 7.3^a^	0.93	0.48 (−0.02, 0.98)	15.9 ± 6.9^a,b^	1.07	**0.79 (0.27**, **1.30)**
Control	21.9 ± 7.4	21.8 ± 8.1	0.01		20.1 ± 9.5	0.24		19.9 ± 8.8	0.27		21.2 ± 8.4	0.09	
MFIS COGN													
Intervention	17.5 ± 5.6	16.6 ± 8.0	0.16	−0.07 (−0.57, 0.42)	14.2 ± 9.3	0.59	0.48 (−0.02, 0.99)	13.6 ± 8.3^a^	0.70	0.28 (−0.21, 0.78)	12.1 ± 8.2^a^	0.96	**0.56 (0.05**, **1.06)**
Control	16.0 ± 7.9	14.6 ± 8.3	0.18		16.1 ± 9.0	−0.01		14.1 ± 9.5	0.24		14.5 ± 9.4	0.19	
MFIS PSYCH													
Intervention	4.5 ± 1.5	3.4 ± 1.8^a^	0.73	0.48 (−0.02, 0.99)	3.3 ± 1.9^a^	0.80	**0.53 (0.03**, **1.04)**	3.1 ± 2.0^a^	0.93	**0.53 (0.03**, **1.04)**	3.0 ± 2.0^a^	1.00	**0.63 (0.12**, **1.14)**
Control	4.1 ± 2.4	4.0 ± 2.2	0.04		4.0 ± 2.4	0.04		3.8 ± 2.3	0.12		3.9 ± 2.4	0.08	

CI: confidence interval; WK: week; STAI-Y2: Trait Subscale of the State-Trait Anxiety Inventory; HADS-A: Anxiety Subscale of the Hospital Anxiety and Depression Scale; QIDS: Quick Inventory of Depressive Symptomatology; HADS-D: Depression Subscale of the Hospital Anxiety and Depression Scale; MFIS total: Modified Fatigue Impact Scale total score; MFIS PHYS: Physical Subscale of the Modified Fatigue Impact Scale; MFIS COGN: Cognitive Subscale of the Modified Fatigue Impact Scale; MFIS PSYCH: Psychosocial Subscale of the Modified Fatigue Impact Scale.

Bold Hedges’ *d* effect sizes are statistically significant based on 95% CI not encompassing 0.

a A statistically significant difference from baseline (*p* < 0.05).

b A statistically significant difference from Control (*p* < 0.05).

### Overall sample

#### Depressive symptoms

For QIDS, the group × time interaction was significant (*F*_(2.888, 219.475)_ = 5.13, *p* ⩽ 0.002). Pilates significantly reduced depressive symptoms between baseline and weeks 6 (*M*_diff_ = −2.97, *p* ⩽ 0.002) and 8 (*M*_diff_ = −3.73, *p* *<* 0.001). Compared to WL, depressive symptoms were significantly lower for Pilates at week 8 (*M*_diff_ = −2.53, *p* ⩽ 0.001, *d* = 0.70, 95% CI: 0.25, 1.15). The mean reduction in depressive symptoms among Pilates participants resulted in NNT = 4 (95% CI: 2, 8). For HADS-D, the group × time interaction was significant (*F*_(3.711, 282.055)_ = 10.12, *p* *<* 0.001). Pilates significantly reduced depressive symptoms between baseline and weeks 2 (*M*_diff_ = −1.24, *p* ⩽ 0.001), 4 (*M*_diff_ = −2.08, *p* *<* 0.001), 6 (*M*_diff_ = −2.58, *p* *<* 0.001) and 8 (*M*_diff_ = −2.93, *p* *<* 0.001). Compared to WL, depressive symptoms were significantly lower for Pilates at week 8 (*M*_diff_ = −1.52, *p* ⩽ 0.03, *d* = 0.74, 95% CI: 0.29, 1.20). The mean reduction in depressive symptoms among Pilates participants resulted in NNT = 3 (95% CI: 2, 7).

### Anxiety symptoms

The group × time interaction for STAI-Y2 was significant (*F*_(3.532, 268.444)_ = 4.19, *p* ⩽ 0.004). Pilates significantly reduced anxiety symptoms between baseline and weeks 4 (*M*_diff_ = −4.11, *p* < 0.03), 6 (*M*_diff_ = −4.92, *p* ⩽ 0.002) and 8 (*M*_diff_ = −6.17, *p* < 0.001). The mean reduction in anxiety symptoms resulted in NNT = 7 (95% CI: −14, 3).

For HADS-A, the group × time interaction was significant (*F*_(3.819, 290.267)_ = 6.87, *p* *<* 0.001). Pilates significantly reduced anxiety symptoms between baseline and weeks 4 (*M*_diff_ = −2.04, *p* < 0.001), 6 (*M*_diff_ = −2.56, *p* < 0.001) and 8 (*M*_diff_ = −3.48, *p* < 0.001). The mean reduction in anxiety symptoms among Pilates participants resulted in NNT = 5 (95% CI: 2, 40).

### Physical symptoms of fatigue

The group × time interaction was significant (*F*_(3.546, 269.474)_ = 4.43, *p* ⩽ 0.003). Pilates significantly reduced physical fatigue between baseline and weeks 2 (*M*_diff_ = −2.92, *p* ⩽ 0.001), 4 (*M*_diff_ = −2.92, *p* *<* 0.03), 6 (*M*_diff_ = −5.21, *p* *<* 0.001) and 8 (*M*_diff_ = −5.92, *p* *<* 0.001). Compared to WL, physical fatigue was significantly lower for Pilates at week 8 (*M*_diff_ = −5.39, *p* ⩽ 0.002, *d* = 0.78, 95% CI: 0.32, 1.23). The mean reduction in physical fatigue resulted in NNT = 3 (95% CI: 2, 6).

### Cognitive symptoms of fatigue

The group × time interaction was significant (*F*_(3.369, 256.071)_ = 4.18, *p* ⩽ 0.005). Pilates significantly reduced cognitive fatigue between baseline and weeks 6 (*M*_diff_ = −3.86, *p* *<* 0.001) and 8 (*M*_diff_ = −5.20, *p* *<* 0.001). The mean reduction in cognitive fatigue resulted in NNT = 5 (95% CI: −200, 2).

### Psychosocial symptoms of fatigue

The group × time interaction was significant (*F*_(3.618, 274.972)_ = 4.08, *p* ⩽ 0.004). Pilates significantly reduced psychosocial fatigue between baseline and weeks 2 (*M*_diff_ = −1.10, *p* ⩽ 0.001), 4 (*M*_diff_ = −1.17, *p* < 0.001), 6 (*M*_diff_ = −1.42, *p* < 0.001) and 8 (*M*_diff_ = −1.53, *p* < 0.001). Compared to WL, psychosocial fatigue was significantly lower for Pilates at week 8 *(M*_diff_ = −1.06, *p* ⩽ 0.02, *d* = 0.56, 95% CI: 0.11, 1.01). The mean reduction in psychosocial fatigue resulted in NNT = 4 (95% CI: 2, 18).

### Total fatigue

The group × time interaction was significant (*F*_(3.423, 260.116)_ = 4.44, *p* ⩽ 0.003). Pilates significantly reduced total fatigue between baseline and weeks 2 (*M*_diff_ = −4.78, *p* ⩽ 0.006), 4 (*M*_diff_ = −7.18, *p* ⩽ 0.002), 6 (*M*_diff_ = −10.51, *p* < 0.001) and 8 (*M*_diff_ = −12.69, *p* < 0.001). Compared to WL, total fatigue was significantly lower for Pilates at week 8 (*M*_diff_ = −9.51, *p* ⩽ 0.007, *d* = 0.76, 95% CI: 0.31, 1.22). The mean reduction in total fatigue resulted in NNT = 3 (95% CI: 2, 6).

### Completers

Results were materially the same for all outcomes (Table 3).

### Female-only sample (ITT)

#### Depressive symptoms

For QIDS, the group × time interaction was significant (*F*_(2.716, 176.536)_ = 3.77, *p* *<* 0.02). Pilates significantly reduced depressive symptoms between baseline and weeks 6 (*M*_diff_ = −2.98, *p* ⩽ 0.004) and 8 (*M*_diff_ = −3.80, *p* *<* 0.001). Compared to WL, depressive symptoms were significantly lower for Pilates at week 8 (*M*_diff_ = −2.87, *p* ⩽ 0.001, *d* = 0.64, 95% CI: 0.16, 1.13). The mean reduction in depressive symptoms resulted in NNT = 4 (95% CI: 2, 12). For HADS-D, the group × time interaction was significant (*F*_(3.707, 240.986)_ = 6.68, *p* *<* 0.001). Pilates significantly reduced depressive symptoms between baseline and weeks 2 (*M*_diff_ = −1.21, *p* ⩽ 0.003), 4 (*M*_diff_ = −2.09, *p* < 0.001), 6 (*M*_diff_ = −2.40, *p* < 0.001) and 8 (*M*_diff_ = −2.87, *p* < 0.001). The mean reduction in depressive symptoms among Pilates participants resulted in NNT = 3 (95% CI: 2, 8).

### Anxiety symptoms

The group × time interaction for STAI-Y2 was significant (*F*_(3.419, 222.233)_ = 2.73, *p* *<* 0.04). Pilates significantly reduced anxiety symptoms between baseline and weeks 4 (*M*_diff_ = −3.79, *p* < 0.04), 6 (*M*_diff_ = −4.53, *p* < 0.02) and 8 (*M*_diff_ = −6.09, *p* ⩽ 0.002). The mean reduction in anxiety symptoms resulted in NNT = 9 (95% CI: −9, 3). For HADS-A, the group × time interaction was significant (*F*_(3.812, 247.771)_ = 6.33, *p* *<* 0.001). Pilates significantly reduced anxiety symptoms between baseline and weeks 4 (*M*_diff_ = −2.03, *p* < 0.001), 6 (*M*_diff_ = −2.42, *p* < 0.001) and 8 (*M*_diff_ = −3.45, *p* < 0.001). The mean reduction in anxiety symptoms among Pilates participants resulted in NNT = 5 (95% CI: −200, 2).

### Physical symptoms of fatigue

The group × time interaction was significant (*F*_(3.410, 221.660)_ = 5.02, *p* ⩽ 0.001). Pilates significantly reduced physical fatigue between baseline and weeks 2 (*M*_diff_ = −2.81, *p* ⩽ 0.001), 6 (*M*_diff_ = −5.37, *p* *<* 0.001) and 8 (*M*_diff_ = −5.98, *p* *<* 0.001). Compared to WL, physical fatigue was significantly lower for Pilates at week 8 (*M*_diff_ = −5.29, *p* ⩽ 0.004, *d* = 0.86, 95% CI: 0.37, 1.35). The mean reduction in physical fatigue resulted in NNT = 3 (95% CI: 2, 5).

### Cognitive symptoms of fatigue

The group × time interaction was significant (*F*_(3.362, 218.558)_ = 4.20, *p* ⩽ 0.005). Pilates significantly reduced cognitive fatigue between baseline and weeks 6 (*M*_diff_ = −3.70, *p* ⩽ 0.002) and 8 (*M*_diff_ = −5.23, *p* *<* 0.001). Compared to WL, cognitive fatigue was significantly lower for Pilates at week 8 (*M*_diff_ = −4.26, *p* *<* 0.05, *d* = 0.48, 95% CI: 0.01, 0.96). The mean reduction in cognitive fatigue resulted in NNT = 5 (95% CI: 2, 200).

### Psychosocial symptoms of fatigue

The group × time interaction was significant (*F*_(3.758, 244.294)_ = 4.37, *p* ⩽ 0.002). Pilates significantly reduced psychosocial fatigue between baseline and weeks 2 (*M*_diff_ = −1.05, *p* ⩽ 0.001), 4 (*M*_diff_ = −1.12, *p* ⩽ 0.001), 6 (*M*_diff_ = −1.44, *p* *<* 0.001) and 8 (*M*_diff_ = −1.59, *p* *<* 0.001). Compared to WL, psychosocial fatigue was significantly lower for Pilates at week 8 (*M*_diff_ = −1.08, *p* *<* 0.03, *d* = 0.63, 95% CI: 0.15, 1.11). The mean reduction in psychosocial fatigue resulted in NNT = 4 (95% CI: 2, 13).

### Total fatigue

The group × time interaction was significant (*F*_(3.448, 224.130)_ = 5.14, *p* ⩽ 0.001). Pilates significantly reduced total fatigue between baseline and weeks 2 (*M*_diff_ = −4.36, *p* *<* 0.03), 4 (*M*_diff_ = −6.74, *p* ⩽ 0.009), 6 (*M*_diff_ = −10.51, *p* *<* 0.001) and 8 (*M*_diff_ = −12.83, *p* *<* 0.001). Compared to WL, total fatigue was significantly lower for Pilates at week 8 (*M*_diff_ = −10.60, *p* ⩽ 0.007, *d* = 0.81, 95% CI: 0.32, 1.30). The mean reduction in total fatigue resulted in NNT = 3 (95% CI: 2, 6).

### Female-only completers

Results were materially the same for all outcomes (Supplementary Table 2).

## Discussion

Home-based Pilates significantly improved depressive and anxiety symptoms, physical, cognitive, psychosocial and total fatigue among PwMS. These findings may be particularly important given the limited treatment success of CBT,^25^ and pharmacotherapy,^4^ for these debilitating comorbidities among PwMS and practical implications of using Pilates among PwMS for whom traditional exercise modes may be difficult.^1^

Symptom improvements ranged from moderate-to-large effects, and are consistent with previously reported effects of traditional exercise modalities on depressive, anxiety and fatigue symptoms among PwMS.^3,4,26^ The magnitude of improvement is consistent with the reported effect of 8 weeks of supervised or home-based Pilates on depressive and fatigue symptoms among PwMS,^13^ and meta-analytic effects of predominantly supervised Pilates among healthy and chronically ill patients.^7^ The meta-analysis primarily involved female samples,^7^ and the present RCT reflected this predominantly female gender profile. In contrast, recent meta-analytic findings did not report any effect of supervised Pilates on depressive symptoms among relatively small samples among PwMS.^12^

Based on a minimally important difference of 0.5 standard deviations,^27^ and consistent with meta-analytic (all Δ ⩾ 0.93) and feasibility trial (all *d* ⩾ 0.50) effect sizes for depression and fatigue responses to Pilates,^7,13^ the magnitude of depressive (all *d* ⩾ 0.70) and fatigue symptom (*d* ⩾ 0.56) improvements herein represent clinically meaningful effects. These findings extend evidence of potentially clinically meaningful effects of Pilates for balance, muscular strength and functional mobility among PwMS.^12^ Based on suggested minimal clinically important differences of four points on the MFIS,^28^ the 12.6-point reduction following Pilates constitutes three times greater, clinically meaningful improvement in fatigue among PwMS. The 41% reduction in QIDS score following Pilates exceeded the suggested 28.5% change representing minimally important improvement.^29^

The NNT for depressive, anxiety and fatigue symptoms were four, five and three, respectively. These findings suggest that for every four participants to complete this Pilates intervention, one would be expected to show significantly improved depressive symptoms; for every five participants to complete this Pilates intervention, one would be expected to show significantly improved anxiety symptoms; and for every three participants to complete this Pilates intervention, one would be expected to show significantly improved fatigue. These NNT compare favourably to Cochrane review findings for antidepressants compared to placebo for treating depression in chronically ill patients,^30^ including MS (NNT = 6 for studies over 6 weeks), the NNT (3.3) for MS-related fatigue following 16 weeks of CBT compared to control among PwMS^31^ and the NNT (1.6–3.3) for anxiety following 8 weeks of mindfulness training compared to optimal medical care among PwMS.^32^

Anxiety symptoms are often underdiagnosed and undertreated among PwMS.^33^ Among the current sample, most participants reported symptoms lower than the estimated cut-score (51.82) suggested to detect clinically significant symptoms.^34^ Although the magnitude of improvement in anxiety (*d* = 0.30 (STAI-Y2); *d* = 0.49 (HADS-A)) is lower than previously reported effects of supervised Pilates among healthy older and sedentary young female populations,^7^ it is consistent with previously reported effects of exercise training among chronically ill adults.^33^ Furthermore, the 2.8-point reduction for HADS-A exceeded the suggested 1.5-point reduction representing minimally important improvement.^35^

There are several practical strengths and implications for future research and applied practice. Home-based Pilates facilitated nationwide recruitment,^13^ while addressing environmental barriers to exercise participation among PwMS.^1^ Pilates’ floor-based approach lessens balance demands and addresses fear of falling concerns among PwMS.^11,12^ This, along with regular telephone calls, particularly with the home-based group, likely influenced the high compliance, comparable to other home-based interventions;^9^ we further note that differing contact times between the intervention and control groups may partly explain the superior effect in the Pilates group.

Potential limitations include that participants were not recruited based on fatigue, anxiety or depression severity; this powered RCT provides the basis for that examination. Future trials should extend current findings among large samples of PwMS recruited for the presence of elevated anxiety, depressive or fatigue symptoms, and compare home-based Pilates to other exercise modalities of low-to-moderate intensity and other empirically supported treatments (i.e. CBT, pharmacotherapy).

## Conclusion

Home-based Pilates significantly improved anxiety, depressive and fatigue symptoms, including moderate-to-large, clinically meaningful improvements in depressive and fatigue symptoms among PwMS, who were predominantly female. Findings support the potential of home-based Pilates as an alternative low-impact exercise modality to improve mental health among PwMS for whom mobility limitations may hamper traditional exercise participation.

## Supplemental Material

sj-pdf-1-msj-10.1177_13524585211009216 – Supplemental material for Home-based Pilates for symptoms of anxiety, depression and fatigue among persons with multiple sclerosis: An 8-week randomized controlled trial Click here for additional data file. Supplemental material, sj-pdf-1-msj-10.1177_13524585211009216 for Home-based Pilates for symptoms of anxiety, depression and fatigue among persons with multiple sclerosis: An 8-week randomized controlled trial by Karl M Fleming, Susan B Coote and Matthew P Herring in Multiple Sclerosis Journal

sj-pdf-2-msj-10.1177_13524585211009216 – Supplemental material for Home-based Pilates for symptoms of anxiety, depression and fatigue among persons with multiple sclerosis: An 8-week randomized controlled trial Click here for additional data file. Supplemental material, sj-pdf-2-msj-10.1177_13524585211009216 for Home-based Pilates for symptoms of anxiety, depression and fatigue among persons with multiple sclerosis: An 8-week randomized controlled trial by Karl M Fleming, Susan B Coote and Matthew P Herring in Multiple Sclerosis Journal

sj-pdf-3-msj-10.1177_13524585211009216 – Supplemental material for Home-based Pilates for symptoms of anxiety, depression and fatigue among persons with multiple sclerosis: An 8-week randomized controlled trial Click here for additional data file. Supplemental material, sj-pdf-3-msj-10.1177_13524585211009216 for Home-based Pilates for symptoms of anxiety, depression and fatigue among persons with multiple sclerosis: An 8-week randomized controlled trial by Karl M Fleming, Susan B Coote and Matthew P Herring in Multiple Sclerosis Journal

sj-pdf-4-msj-10.1177_13524585211009216 – Supplemental material for Home-based Pilates for symptoms of anxiety, depression and fatigue among persons with multiple sclerosis: An 8-week randomized controlled trial Click here for additional data file. Supplemental material, sj-pdf-4-msj-10.1177_13524585211009216 for Home-based Pilates for symptoms of anxiety, depression and fatigue among persons with multiple sclerosis: An 8-week randomized controlled trial by Karl M Fleming, Susan B Coote and Matthew P Herring in Multiple Sclerosis Journal

sj-pdf-5-msj-10.1177_13524585211009216 – Supplemental material for Home-based Pilates for symptoms of anxiety, depression and fatigue among persons with multiple sclerosis: An 8-week randomized controlled trial Click here for additional data file. Supplemental material, sj-pdf-5-msj-10.1177_13524585211009216 for Home-based Pilates for symptoms of anxiety, depression and fatigue among persons with multiple sclerosis: An 8-week randomized controlled trial by Karl M Fleming, Susan B Coote and Matthew P Herring in Multiple Sclerosis Journal

sj-pdf-6-msj-10.1177_13524585211009216 – Supplemental material for Home-based Pilates for symptoms of anxiety, depression and fatigue among persons with multiple sclerosis: An 8-week randomized controlled trial Click here for additional data file. Supplemental material, sj-pdf-6-msj-10.1177_13524585211009216 for Home-based Pilates for symptoms of anxiety, depression and fatigue among persons with multiple sclerosis: An 8-week randomized controlled trial by Karl M Fleming, Susan B Coote and Matthew P Herring in Multiple Sclerosis Journal
