# Supplementary material for: Home-based Pilates for symptoms of anxiety, depression and fatigue among persons with multiple sclerosis: An 8-week randomized controlled trial
Source: Mult Scler. 2021 Apr 19;27(14):2267–79. doi: 10.1177/13524585211009216 (PMC8597189; doi:10.1177/13524585211009216)
Supplement: sj-pdf-2-msj-10.1177_13524585211009216 – Supplemental material for Home-based Pilates for symptoms of anxiety, depression and fatigue among persons with multiple sclerosis: An 8-week randomized controlled trial [file sj-pdf-2-msj-10.1177_13524585211009216.pdf]

Supplemental Materials

Title – "Supplementary Table 1- ITT Primary Outcomes Females 30OCT2020 - Supplemental material for Home-based Pilates for Symptoms of Anxiety, Depression, and Fatigue among Persons with Multiple Sclerosis"; Description – "Supplemental material, [Supplementary Table 1- ITT Primary Outcomes Females 30OCT2020], for [Home-based Pilates for Symptoms of Anxiety, Depression, and Fatigue among Persons with Multiple Sclerosis: An Eight-week Randomized Controlled Trial] by [Karl M Fleming, Susan B Coote, Matthew P Herring] in [Multiple Sclerosis Journal]".

Title – "Supplementary Table 2 - Primary Outcomes Females Only 30OCT2020 - Supplemental material for Home-based Pilates for Symptoms of Anxiety, Depression, and Fatigue among Persons with Multiple Sclerosis"; Description – "Supplemental material, [Supplementary Table 2 - Primary Outcomes Females Only 30OCT2020], for [Home-based Pilates for Symptoms of Anxiety, Depression, and Fatigue among Persons with Multiple Sclerosis: An Eight-week Randomized Controlled Trial] by [Karl M Fleming, Susan B Coote, Matthew P Herring] in [Multiple Sclerosis Journal]".

Title – "Supplementary Table 3 – Symptom Classifications 20MAR2021 - Supplemental material for Home-based Pilates for Symptoms of Anxiety, Depression, and Fatigue among Persons with Multiple Sclerosis"; Description – "Supplemental material, [Supplementary Table 3 - Symptom Classifications 20MAR2021], for [Home-based Pilates for Symptoms of Anxiety, Depression, and Fatigue among Persons with Multiple Sclerosis: An Eight-week Randomized Controlled Trial] by [Karl M Fleming, Susan B Coote, Matthew P Herring] in [Multiple Sclerosis Journal]".

Title – "Supplementary Table 4 – Point-Biserial Correlations 20MAR2021 - Supplemental material for Home-based Pilates for Symptoms of Anxiety, Depression, and Fatigue among Persons with Multiple Sclerosis"; Description – "Supplemental material, [Supplementary Table 4 – Point-Biserial Correlations 20MAR2021], for [Home-based Pilates for Symptoms of Anxiety, Depression, and Fatigue among Persons with Multiple Sclerosis: An Eight-week Randomized Controlled Trial] by [Karl M Fleming, Susan B Coote, Matthew P Herring] in [Multiple Sclerosis Journal]".
