# Supplementary material for: Home-based Pilates for symptoms of anxiety, depression and fatigue among persons with multiple sclerosis: An 8-week randomized controlled trial
Source: Mult Scler. 2021 Apr 19;27(14):2267–79. doi: 10.1177/13524585211009216 (PMC8597189; doi:10.1177/13524585211009216)
Supplement: sj-pdf-3-msj-10.1177_13524585211009216 – Supplemental material for Home-based Pilates for symptoms of anxiety, depression and fatigue among persons with multiple sclerosis: An 8-week randomized controlled trial [file sj-pdf-3-msj-10.1177_13524585211009216.pdf]

Supplementary Table 1. ITT Primary outcome changes at each measurement (Female only) - means (SD), within-group magnitude of change and between-group magnitude of differences in change were quantified using standardized mean differences (d) and Hedges' *d* (95%CIs), respectively)

Outcome	Baseline	WK 2			WK 4			WK 6			WK 8		
	Mean ± SD	Mean ± SD	d	Hedges' *d* from baseline	Mean ± SD	d	Hedges' *d* from baseline	Mean ± SD	d	Hedges' *d* from baseline	Mean ± SD	d	Hedges' *d* from baseline
**STAI-Y2**													
Intervention	43.1±9.8	43.4±9.3	-0.03	-0.13 (-0.60, 0.34)	39.6±9.8*	0.36	0.19 (-0.29, 0.66)	38.7±10.2*	0.45	0.24 (-0.23, 0.72)	37.4±9.3*	0.58	0.24 (-0.23, 0.72)
Control	43.4±11.3	42.3±10.1	0.10		41.9±11.4	0.13		41.6±10.9	0.16		40.3±10.1	0.27	
**HADS–A**													
Intervention	8.5±3.8	7.6±3.4	0.24	0.10 (-0.38, 0.57)	6.6±3.4*	0.50	0.29 (-0.19, 0.76)	6.3±2.9*	0.58	0.41 (-0.07, 0.88)	5.3±2.8*	0.84	0.48 (-0.01, 0.96)
Control	7.6±4.5	7.1±3.9	0.11		6.9±4.0	0.16		7.1±4.5	0.11		6.4±4.4	0.27	
**QIDS**													
Intervention	9.0±4.1	7.4±3.3	0.39	0.30 (-0.17, 0.78)	7.2±3.2	0.44	0.34 (-0.13, 0.82)	6.0±3.6*	0.73	0.36 (-0.11, 0.84)	5.3±2.7*§	0.90	**0.64 (0.16, 1.13)**
Control	8.5±5.1	8.3±3.8	0.04		8.3±4.0	0.04		7.2±3.7	0.25		7.8±4.0	0.14	
**HADS-D**													
Intervention	6.9±3.2	5.6±2.9*	0.41	0.24 (-0.23, 0.72)	4.8±2.7*	0.66	0.43 (-0.05, 0.90)	4.4±3.1*	0.78	**0.70 (0.21, 1.19)**	4.0±3.1*	0.91	**0.73 (0.24, 1.22)**
Control	5.8±3.3	5.3±3.2	0.15		5.1±3.2	0.21		5.6±4.1	0.06		5.3±2.9	0.15	
**MFIS Total**													
Intervention	44.0±9.9	39.6±11.7*	0.44	0.23 (-0.24, 0.70)	37.1±14.9*	0.70	0.33 (-0.15, 0.80)	33.7±13.9*	1.04	**0.50 (0.02, 0.98)**	31.4±13.8*§	1.27	**0.81 (0.32, 1.30)**
Control	44.2±14.7	42.7±14.8	0.10		41.4±15.9	0.19		40.2±17.5	0.27		41.8±16.4	0.16	
**MFIS PHYS**													
Intervention	22.1±5.4	19.2±5.2*	0.54	0.46 (-0.02, 0.94)	19.0±6.6	0.57	0.22 (-0.25, 0.70)	16.7±6.6*	1.00	**0.56 (0.08, 1.04)**	16.1±6.3*§	1.11	**0.86 (0.37, 1.35)**
Control	21.8±7.0	21.8±7.8	0.00		20.1±8.6	0.24		19.9±8.5	0.27		21.2±8.0	0.09	
**MFIS COGN**													
Intervention	17.3±6.8	16.8±8.0	0.07	-0.11 (-0.58, 0.37)	14.7±8.9	0.38	0.23 (-0.25, 0.70)	13.8±8.4*	0.51	0.24 (-0.23, 0.72)	12.3±8.2*§	0.74	**0.48 (0.01, 0.96)**
Control	18.1±7.9	16.8±8.0	0.16		17.2±7.7	0.11		16.4±9.1	0.22		16.7±8.9	0.18	
**MFIS PSYCH**													
Intervention	4.6±1.4	3.6±1.6*	0.71	0.42 (-0.06, 0.90)	3.5±1.8*	0.79	0.42 (-0.06, 0.90)	3.2±1.8*	1.00	**0.53 (0.04, 1.01)**	3.1±1.7*§	1.07	**0.63 (0.15, 1.11)**
Control	4.3±2.3	4.1±2.0	0.09		4.0±2.3	0.13		3.9±2.3	0.17		4.0±2.3	0.13	

*A statistically significant difference from baseline ($p<0.05$)

§ A statistically significant difference from Control ($p<0.05$)

Bold Hedges' *d* effect sizes are statistically significant based on 95%CI not encompassing 0

Abbreviations: HADS-A: Anxiety Subscale of the Hospital Anxiety and Depression Scale; HADS-D: Depression Subscale of the Hospital Anxiety and Depression Scale; ITT: Intention to treat; MFIS COGN: Cognitive Subscale of the Modified Fatigue Impact Scale; MFIS PHYS: Physical Subscale of the Modified Fatigue Impact Scale; MFIS PSYCH: Psychosocial Subscale of the Modified Fatigue Impact Scale; MFIS Total: Modified Fatigue Impact Scale total score; QIDS: Quick Inventory of Depressive Symptomatology; SD: Standard deviation; SMD: Standardised mean difference; STAI-Y2: Trait Subscale of the State-Trait Anxiety Inventory; WK: Week.
