# Supplementary material for: Home-based Pilates for symptoms of anxiety, depression and fatigue among persons with multiple sclerosis: An 8-week randomized controlled trial
Source: Mult Scler. 2021 Apr 19;27(14):2267–79. doi: 10.1177/13524585211009216 (PMC8597189; doi:10.1177/13524585211009216)
Supplement: sj-pdf-4-msj-10.1177_13524585211009216 – Supplemental material for Home-based Pilates for symptoms of anxiety, depression and fatigue among persons with multiple sclerosis: An 8-week randomized controlled trial [file sj-pdf-4-msj-10.1177_13524585211009216.pdf]

Supplementary Table 2. Primary outcome changes at each measurement - means (SD), within-group magnitude of change and between-group magnitude of differences in change were quantified using standardized mean differences (d) and Hedges' *d* (95%CIs), respectively) (Female-only)

Outcome	Baseline	WK 2			WK 4			WK 6			WK 8		
	Mean ± SD	Mean ± SD	d	Hedges' *d* from baseline	Mean ± SD	d	Hedges' *d* from baseline	Mean ± SD	d	Hedges' *d* from baseline	Mean ± SD	d	Hedges' *d* from baseline
**STAI-Y2**													
Intervention	43.4±10.8	43.4±9.8	0.00	-0.06 (-0.59, 0.47)	40.1±10.9	0.30	0.18 (-0.36, 0.71)	39.0±11.4	0.41	0.26 (-0.27, 0.80)	37.3±10.4	0.57	0.31 (-0.23, 0.84)
Control	42.9±11.4	42.2±11.0	0.06		41.7±12.4	0.11		41.4±11.8	0.13		40.3±11.1	0.23	
**HADS–A**													
Intervention	8.6±3.8	7.4±3.3	0.32	0.20 (-0.34, 0.73)	6.6±3.6*	0.53	0.35 (-0.19, 0.89)	6.2±3.0*	0.63	0.50 (-0.04, 1.04)	5.1±2.8*	0.92	**0.62 (0.07, 1.17)**
Control	7.3±4.1	6.9±4.1	0.10		6.7±4.1	0.15		6.9±4.8	0.10		6.3±4.7	0.24	
**QIDS**													
Intervention	8.8±4.3	7.4±3.3	0.32	0.25 (-0.28, 0.79)	7.3±3.6	0.36	0.33 (-0.21, 0.86)	5.9±4.2*	0.66	0.39 (-0.15, 0.93)	5.1±3.1*§	0.85	**0.69 (0.14, 1.24)**
Control	8.2±4.9	8.0±4.0	0.04		8.1±4.4	0.01		7.1±4.0	0.21		7.7±4.3	0.09	
**HADS-D**													
Intervention	7.0±3.6	5.7±3.2*	0.36	0.29 (-0.25, 0.82)	4.9±3.0*	0.58	0.43 (-0.11, 0.97)	4.6±3.5*	0.67	**0.63 (0.08, 1.17)**	4.0±3.6*	0.83	**0.77 (0.22, 1.32)**
Control	5.4±3.3	5.1±3.4	0.10		4.8±3.4	0.18		5.2±4.3	0.06		5.1±3.1	0.10	
**MFIS Total**													
Intervention	43.8±9.9	39.9±12.6*	0.40	0.23 (-0.30, 0.77)	36.7±16.4*	0.71	0.37 (-0.17, 0.90)	33.4±15.3*	1.06	0.53 (-0.01, 1.07)	31.1±15.4*§	1.28	**0.84 (0.29, 1.40)**
Control	42.5±15.7	41.7±16.0	0.05		40.3±17.2	0.14		39.1±19.1	0.21		41.0±17.9	0.09	
**MFIS PHYS**													
Intervention	21.7±5.5	19.2±5.7*	0.45	0.41 (-0.13, 0.94)	18.6±7.2*	0.56	0.24 (-0.29, 0.78)	16.3±7.3*	0.98	**0.57 (0.02, 1.11)**	15.7±7.0*§	1.08	**0.87 (0.31, 1.42)**
Control	21.6±7.3	21.8±8.2	-0.03		20.1±9.2	0.20		20.0±9.0	0.22		21.3±8.5	0.04	
**MFIS COGN**													
Intervention	17.7±5.2	17.2±7.7	0.09	-0.09 (-0.62, 0.45)	14.8±9.2*	0.55	0.34 (-0.20, 0.88)	14.0±8.2*	0.70	0.32 (-0.22, 0.85)	12.5±8.3*	0.99	**0.60 (0.06, 1.15)**
Control	16.9±8.0	15.8±8.4	0.13		16.4±8.0	0.07		15.4±9.7	0.19		15.8±9.4	0.13	
**MFIS PSYCH**													
Intervention	4.4±1.4	3.4±1.8*	0.67	0.51 (-0.04, 1.05)	3.3±2.0*	0.77	0.48 (-0.06, 1.02)	3.0±2.0*	0.94	**0.58 (0.03, 1.12)**	2.9±1.9*	1.05	**0.69 (0.14, 1.23)**
Control	4.0±2.4	4.0±2.2	0.08		3.8±2.4	0.06		3.8±2.5	0.09		3.8±2.5	0.06	

*A statistically significant difference from baseline (p<0.05)

§ A statistically significant difference from Control (p<0.05)

Bold Hedges' *d* effect sizes are statistically significant based on 95%CI not encompassing 0

Abbreviations: HADS-A: Anxiety Subscale of the Hospital Anxiety and Depression Scale; HADS-D: Depression Subscale of the Hospital Anxiety and Depression Scale; MFIS COGN: Cognitive Subscale of the Modified Fatigue Impact Scale; MFIS PHYS: Physical Subscale of the Modified Fatigue Impact Scale; MFIS PSYCH: Psychosocial Subscale of the Modified Fatigue Impact Scale; MFIS Total: Modified Fatigue Impact Scale total score; QIDS: Quick Inventory of Depressive Symptomatology; SD: Standard deviation; SMD: Standardised mean difference; STAI-Y2: Trait Subscale of the State-Trait Anxiety Inventory; WK: Week.
