# Supplementary material for: Home-based Pilates for symptoms of anxiety, depression and fatigue among persons with multiple sclerosis: An 8-week randomized controlled trial
Source: Mult Scler. 2021 Apr 19;27(14):2267–79. doi: 10.1177/13524585211009216 (PMC8597189; doi:10.1177/13524585211009216)
Supplement: sj-pdf-5-msj-10.1177_13524585211009216 – Supplemental material for Home-based Pilates for symptoms of anxiety, depression and fatigue among persons with multiple sclerosis: An 8-week randomized controlled trial [file sj-pdf-5-msj-10.1177_13524585211009216.pdf]

Supplementary Table 3. Number of participants with clinical symptom severity at baseline

	ITT Full Sample			All Completers			ITT Female Only			Female Only Completers		
**Classification**	**H/B (n=39)**	**WL (n=41)**	**Total (n=80)**	**H/B (n=29)**	**WL (n=34)**	**Total (n=63)**	**H/B (n=36)**	**WL (n=33)**	**Total (n=69)**	**H/B (n=27)**	**WL (n=27)**	**Total (n=80)**
**Single**												
Depressed (QIDS)	32	24	56	23	20	43	30	22	52	21	18	39
Depressed (HADS-D)	14	11	25	11	7	18	13	9	22	10	5	15
Anxious (HADS-A)	22	17	39	17	14	31	21	16	37	16	13	29
Hi-trait Anxious (STAI-Y2)	5	8	13	4	7	11	5	8	13	4	7	11
Fatigued (MFIS)	27	28	55	21	21	42	25	23	48	8	10	18
**Double**												
QIDS/HADS-A	20	12	32	15	10	25	19	12	31	14	10	24
QIDS/STAI-Y2	5	8	13	4	7	11	5	8	13	4	7	11
HADS-D/HADS-A	9	7	16	6	4	10	9	7	16	6	4	10
HADS-D/STAI-Y2	4	4	8	3	3	6	4	4	8	3	3	6
QIDS/MFIS	25	19	44	19	15	34	23	18	41	17	14	31
HADS-D/MFIS	12	9	21	9	5	14	11	8	19	8	4	12
HADS-A/MFIS	18	13	31	13	10	23	17	12	29	12	9	21
MFIS/STAI-Y2	4	8	12	3	7	10	4	8	12	3	7	10
**Treble**												
QIDS/HADS-A/MFIS	17	10	27	12	8	20	16	10	26	11	8	19
QIDS/STAI-Y2/MFIS	4	8	12	3	7	10	4	8	12	3	7	10
HADS-D/HADS-A/MFIS	8	6	5	3	8	24	8	6	14	5	3	8
HADS-D/STAI-Y2/MFIS	4	4	8	3	3	6	4	4	8	3	3	6

Abbreviations: HADS-A: Anxiety Subscale of the Hospital Anxiety and Depression Scale; HADS-D: Depression Subscale of the Hospital Anxiety and Depression Scale; H/B: Home-based intervention group; ITT: Intention to treat; MFIS: Modified Fatigue Impact Scale total score; QIDS: Quick Inventory of Depressive Symptomatology; STAI-Y2: Trait Subscale of the State-Trait Anxiety Inventory; WL: Wait-list control.
