# Supplementary material for: Home-based Pilates for symptoms of anxiety, depression and fatigue among persons with multiple sclerosis: An 8-week randomized controlled trial
Source: Mult Scler. 2021 Apr 19;27(14):2267–79. doi: 10.1177/13524585211009216 (PMC8597189; doi:10.1177/13524585211009216)
Supplement: sj-pdf-6-msj-10.1177_13524585211009216 – Supplemental material for Home-based Pilates for symptoms of anxiety, depression and fatigue among persons with multiple sclerosis: An 8-week randomized controlled trial [file sj-pdf-6-msj-10.1177_13524585211009216.pdf]

Supplementary Table 4. Point-Biserial correlations (r_{pb}) between baseline symptom severity classification and intervention outcome change between baseline and week eight

Outcome	ITT FULL SAMPLE			ALL COMPLETERS			ITT FEMALE ONLY			FEMALE ONLY COMPLETERS		
	**H/B**	**WL**	**TOTAL**	**H/B**	**WL**	**TOTAL**	**H/B**	**WL**	**TOTAL**	**H/B**	**WL**	**TOTAL**
QIDS	0.76*	0.70*	0.71*	0.73*	0.62*	0.66*	0.75*	0.68*	0.69*	0.73*	0.59*	0.64*
HADS-D	0.38	0.35	0.34*	0.33	0.27	0.31	0.40	0.46	0.39*	0.36	0.37	0.38
HADS-A	0.58*	0.29	0.48*	0.56*	0.16	0.43*	0.56*	0.30	0.46*	0.54*	0.16	0.41*
STAI-Y2	0.35	0.49*	0.40*	0.37	0.42	0.36*	0.37	0.49	0.40*	0.40	0.43	0.37
MFIS	0.19	0.24	0.20	0.17	0.21	0.22	0.19	0.17	0.16	0.17	0.13	0.17

***p**<0.0033 (adjusted for multiple testing)

Abbreviations: HADS-A: Anxiety Subscale of the Hospital Anxiety and Depression Scale; HADS-D: Depression Subscale of the Hospital Anxiety and Depression Scale; ITT: Intention to treat; MFIS: Modified Fatigue Impact Scale total score; QIDS: Quick Inventory of Depressive Symptomatology; r_{pb}: Point-biserial Correlation; STAI-Y2: Trait Subscale of the State-Trait Anxiety Inventory.
